# Supplementary material for: The Hunger Games: Homeostatic State-Dependent Fluctuations in Disinhibition Measured with a Novel Gamified Test Battery
Source: Nutrients. 2021 Jun 10;13(6):2001. doi: 10.3390/nu13062001 (PMC8230368; doi:10.3390/nu13062001)
Supplement: Supplementary file 1 [file nutrients-13-02001-s001.zip › nutrients-1240513-supplementary.pdf]

Supplementary Information

Supplementary Methods

Participants

Table S1. *Demographic information of participants*

	M (SD)	Range
Age (years)	26.26 (6.85)	18-40
Education (years)	14.26 (4.01)	0.5-19
Gender (Female / Male)	20 (59%) / 14 (41%)	
BMI (kg/m^2)	22.84 (3.89)	17.10-35.55
Waist Circumference (cm)	80.34 (12.99)	62-123
Hip Circumference (cm)	96.40 (8.57)	81-115

Note: M, Mean; SD, Standard deviation; BMI, Body mass index.

Drift Diffusion Modelling defining information gathering during the Caravan Spotter task

We further classified performance during this task using the Drift Diffusion Modelling framework [1]. Information-accumulation traditional drift diffusion model assumes that the participant's response times are drawn from a Wiener first passage time process:

$$RT \sim wfpt(\alpha, \tau, z, v)$$

This process contains four parameters of the model: the drift rate v, the boundary separation a, the prior decision bias z and the non-decision time τ. The drift rate corresponds to information accumulation (v), which can be interpreted as a general measure of sensitivity to the relevant configurations. The boundary separation (α) can be interpreted as a decision threshold reflecting the amount of information required to trigger the corresponding choice. The mean 'non-decision' time parameter (τ) refers to the time taken for all processes occurring

prior to (i.e., sensory encoding) and after the decision-making process (i.e., motor-response) . The starting point bias (z) describes whether there is an evidence accumulation advantage to the correct response. We applied the Hierarchical Drift–Diffusion Modelling toolbox to obtain the parameters from the data (HDDM; [2]). This model assumes that the behaviour response times are drawn from group-level distributions, and uses Bayesian statistical methods to estimate the robust parameters from a group and subject level (see [2] for the informative priors chosen in this model).

We compared the drift diffusion model through different parameter constraints. The first model assumes an unbiased starting point (z=0.5). The second model assumes variable starting points across participants and different drift rates. Each model parameter had individual-level distributions for each participant. For each model, we generated 7000 samples from the joint posterior distribution of all model parameters by using Markov chain Monte Carlo methods. The initial 5000 samples were discarded as burn-in to minimize the effect of initial values on the posterior inference (see [2] for more details of the procedure). Geweke statistic was used to assess the convergence of the Markov chains.

Signal Detection Theory defining task decision-making during the Bounty Hunter task

Behavioural performance during the Bounty Hunter task of the CIS was described within the Signal Detection Theory framework [3], which discriminates between two parameters: (1) sensitivity (d') and (2) response criterion (c). These parameters were computed as follows using Matlab (R2018b):

(1) Sensitivity d':

For normally distributed signal and noise with mean and standard deviations μ_S and σ_S, and μ_N and σ_N, respectively, d' is defined as:

$$d' = \frac{\mu_S - \mu_S}{\sqrt{\frac{1}{2}(\sigma_S^2 + \sigma_N^2)}}$$

d′ assumes that the standard deviations for signal and noise are equal. *d′* can be estimated from the observed hit rate and false alarm rates, as follows:

$$d' = z(hits) - z(false\ alarms)$$

where function $z(p)$, $p \in [0,1]$, is the inverse of the cumulative distribution function of the Gaussian distribution.

(2) Criterion c:

The common change in z(False alarms) and z(Hits) reflects a criterion shift, given by the position of the midpoint between z(False alarms) and z(Hits). Thus:

$$c = -\frac{[z(hits) + z(false\ alarms)]}{2}.$$

Blood Drawing Protocol

Verbal consent was obtained prior to all cannulation procedures. Phlebotomists first measured vital signs (i.e., blood pressure, heart rate, respiratory rate, oral temperature) to confirm suitability for cannulation. Acceptable vital sign ranges were based on a standard risk management protocol in the B.A.S.E. facility. Participants with vitals beyond acceptable ranges were re-assessed and rescheduled if necessary. Phlebotomists next assessed for participant state anxiety. A relaxation protocol was available during cannulation for distressed participants, which involved listening to pre-recorded music consistent with

intervention recommendations to decrease state anxiety (e.g., non-lyrical, 60-80 beats per minute;[4]).

Phlebotomists collected 4ml of blood at each blood draw. Samples were centrifuged for 10 minutes at 4C and 27,000rpm to separate plasma and stored at -80C until processing for leptin, LEAP-2 and insulin. For processing, samples were defrosted on ice and measured in duplicates. Following ELISAs were used: LEAP-2 (Phoenix Pharmaceuticals, Catalog #EK-075-40), Insulin (Chrystal Chem, Catalog #90095), Leptin (Invitrogen, Catalog #KAC2281). The optical density (OD) values were obtained spectrophotometrically with a reader (SPECTRAmaxPLUS384) and the average OD produced for duplicate standards was used to create a standard curve. The results were calculated using the standard curve.

Raw Data Cleaning

Quality checks on this final data set were performed to ensure validity of cognitive data using Eisenberg et al.'s [5] criteria. On each CIS task, data was removed if participants took on average less than 200ms to respond on each trial or more than 3,000ms to complete each task. For Bounty Hunter, data was removed if the total proportion of correct responses was less than 82% (z-score 2.5) or if the proportion of correct responses on go trials (preceded by a go cue) was less than 25%. For Caravan Spotter, data was removed if the total proportion of correct hits was less than 69% (z-score 2.5), if participants failed to respond to over 25% of trials or if participants responded using only left or right keys for over 95% of trials. For Prospectors Gamble, data was removed if the proportion of correct responses was less than 47% (z-score 2.5) or if participants responded using only left or right keys for over 95% of trials. Failing quality checks led to removal of participant data from the particular task, but not from other tasks where the participant performed within the expected range.

Supplementary Results

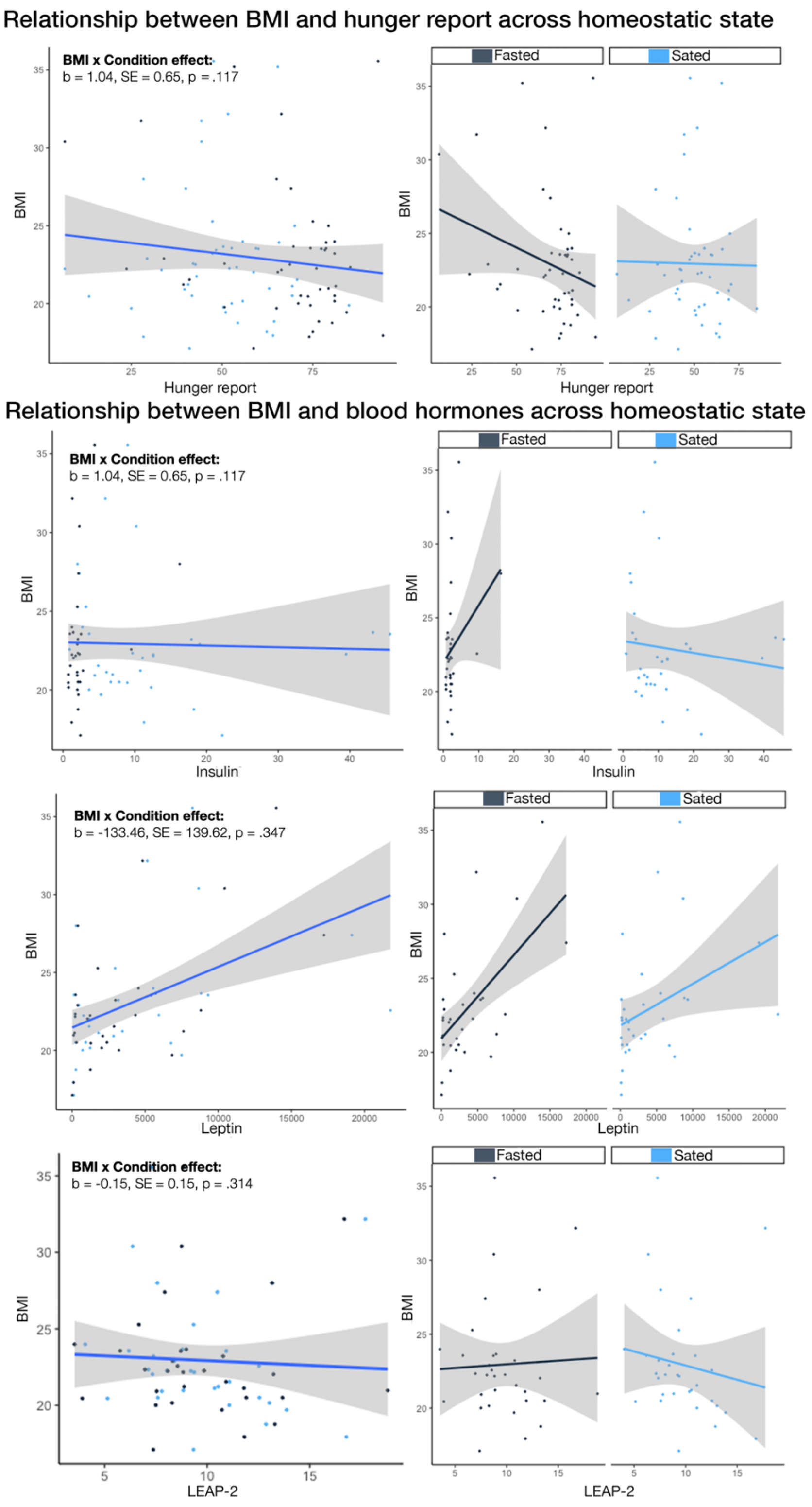

Figure S1. Relationship between BMI and hunger report as well as BMI and blood hormones across homeostatic state.

Table S2. Differences in subjective reports and blood hormones across homeostatic state.

		Fasted		Sated		Paired samples t statistics [c]
		Mean (SD)	Range	Mean (SD)	Range	
Subjective Reports	Hunger	68.44 (19.02)	6.67-94.33	47.47 (15.03)	6.67-70.67	$t(31) = 6.12$, $p < 0.001$
	Anxiety	36.41 (24.63)	0-88.67	36.18 (21.96)	0-68.33	$t(31) = 0.06$, $p = 0.95$
	Mood	61.02 (12.88)	34.33-93	60.11 (14.43)	29-99	$t(31) = 0.38$, $p = 0.70$
Blood Hormones	Insulin mU/L	2.55 (2.99)	0.73-16.27	11.65 (11.51)	0.89-45.58	t(31) = -4.09, $p < 0.001$
	LEAP-2 ng/ml	9.73 (3.34)	3.54-18.80	9.69 (3.13)	4.05-17.71	t(31) = 0.06, $p < 0.95$
	Leptin pg/ml	3666.11 (4250.89)	12.96-17228.48	4149.50 (5343.21)	100-21774.75	t(31) = -0.86, $p < 0.40$

[a] Subjective reports were assessed on a visual analogue scale ranging from 0 to 100. Hunger: Please rate the degree of hunger that you feel right now; Anxiety: Please rate the degree of physical anxiety that you feel right now; Mood: Please rate your current mood.

[C] Bonferroni-corrected significance for three tests related to subjective reports and blood hormones.

Table S3. Differences blood hormones at the start and end of each experimental session

Blood hormones	Start				End			
	Fasted		Sated		Fasted		Sated	
	Mean (SD)	Range	Mean (SD)	Range	Mean (SD)	Range	Mean (SD)	Range
Insulin mU/L	2.08 (1.29)	0.73-16.27	2.13 (1.12)	0.67-6.01	2.55 (2.99)	0.73-16.27	11.65 (11.51)	0.89-45.58
LEAP-2 ng/ml	9.11 (4.00)	3.54-18.80	8.95 (2.88)	3.86-14.63	9.73 (3.34)	3.54-18.80	9.69 (3.13)	4.05-17.71
Leptin pg/ml	4500.33 (4521.22)	12.96-17228.48	4376.90 (5001.67)	100-21774.75	3666.11 (4250.89)	12.96-17228.48	4149.50 (5343.21)	100-21774.75

Table S4. Linear Mixed Effects Modelling for Total Reaction Time.

	Total RT		RT correct responses		RT for errors	
	Full Model	Final Model	Full Model	Final Model	Full Model	Final Model
(Intercept)	0.54***	0.54 ***	0.55 ***	0.55 ***	0.49 ***	0.50 ***
	(0.02)	(0.01)	(0.02)	(0.01)	(0.02)	(0.01)
Condition	-0.02	-0.01	-0.02	-0.01	-0.01	-0.01
	(0.01)	(0.01)	(0.01)	(0.01)	(0.02)	(0.01)
Hunger	0.01		0.01		0.01	
	(0.01)		(0.01)		(0.01)	
Insulin	0.01		0.01		-0.00	
	(0.03)		(0.02)		(0.02)	
Leptin	-0.01		0.00		-0.00	
	(0.01)		(0.01)		(0.01)	
LEAP-2	0.00	0.02	0.01	0.01	0.01	0.00
	(0.01)	(0.01)	(0.01)	(0.01)	(0.01)	(0.01)
Condition x Hunger	-0.03		-0.02		-0.04*	
	(0.01)		(0.01)		(0.01)	

Condition x Insulin	-0.01		-0.01		0.01	
	(0.02)		(0.02)		(0.01)	
Condition x Leptin	0.01		0.01		0.01	
	(0.01)		(0.01)		(0.01)	
Condition x LEAP-2	-0.03 *	-0.03 **	-0.03 *	-0.03 **	-0.02*	-0.04**
	(0.01)	(0.01)	(0.01)	(0.01)	(0.01)	(0.01)
AIC	-81.71	-134.41	-82.74	-135.83	-81.20	-128.04
BIC	-59.51	-123.31	-60.53	-124.73	-59.00	-116.94
Log Likelihood	52.86	73.21	53.37	73.92	52.60	70.02

Note: *AIC*, Akaike information criterion; *BIC*, Bayesian information criterion. Conditions are treatment coded (0 = Fasted, 1 = Sated). *** $p < 0.001$, * $p < 0.05$

Supplementary References

1. Ratcliff R, McKoon G. The Diffusion Decision Model: Theory and Data for Two-Choice Decision Tasks. Neural Computation. 2008;20(4):873-922.
2. Wiecki T, Sofer I, Frank M. HDDM: Hierarchical Bayesian estimation of the Drift-Diffusion Model in Python. Frontiers in Neuroinformatics. 2013;7(14).
3. Macmillan NA, Creelman CD. Detection theory: A user's guide. Cambridge University Press: New York, NY, US; 1991.
4. Nilsson U. The Anxiety- and Pain-Reducing Effects of Music Interventions: A Systematic Review. AORN J. 2008; 87:780–807.
5. Eisenberg IW, Bissett PG, Zeynep Enkavi A, Li J, MacKinnon DP, Marsch LA, et al. Uncovering the structure of self-regulation through data-driven ontology discovery. Nature Communications. 2019;10(1):2319.
